# Verbal modeling, counterconditioning, and operant conditioning are effective in nocebo hyperalgesia attenuation

**DOI:** 10.1097/j.pain.0000000000003934

**Published:** 2026-03-04

**Authors:** Daryna Rubanets, Izabela Łaska, Joanna Kłosowska, Przemysław Bąbel, Elżbieta Anita Bajcar

**Affiliations:** a Pain Research Group, Jagiellonian University, Institute of Psychology, Kraków, Poland; b Doctoral School in the Social Sciences, Jagiellonian University, Kraków, Poland

**Keywords:** Nocebo hyperalgesia, Nocebo effect, Classical conditioning, Counterconditioning, Operant conditioning, Observational learning, Expectancy

## Abstract

Nocebo hyperalgesia, the increased pain following inert interventions, is frequently observed in clinical practice. This study investigates whether nocebo hyperalgesia can be reduced through counterconditioning, verbal modeling, and operant conditioning. Healthy volunteers (N = 168) were randomly allocated to 3 experimental and 2 control groups. In the experimental groups, nocebo hyperalgesia was induced through classical conditioning by applying high-intensity pain stimuli with a placebo and low-intensity pain stimuli without a placebo. In the control groups, sham conditioning was implemented (the relation between pain intensity and placebo was noncontingent). Nocebo hyperalgesia was then attenuated by (1) counterconditioning (low-intensity pain stimuli with a placebo, and high-intensity pain stimuli without a placebo); (2) verbal modeling (moderate pain stimuli with and without a placebo, with pain ratings provided by others suggesting less pain with placebo); (3) operant conditioning (moderate pain stimuli with and without a placebo, and participants were rewarded when experiencing less pain with placebo). In one control group, sham conditioning was continued; the other group received no manipulation. Classical conditioning induced nocebo hyperalgesia. All 3 learning procedures effectively attenuated the nocebo effect with no difference in their effectiveness. The induction of nocebo hyperalgesia was fully mediated by expectancies, whereas the attenuation of this effect was mediated by expectancies only when elicited through operant conditioning. This study demonstrates that nocebo hyperalgesia can be attenuated by verbal modeling, counterconditioning, and operant conditioning. Our findings suggest that healthcare workers may have flexibility in selecting nocebo-attenuating procedures when considering the treatment context or patient preferences.

## Abstract 2

Supplemental Digital Content is Available in the Text.

Verbal modeling, counterconditioning, and operant conditioning equally reduced nocebo hyperalgesia. Expectancies mediated induction, but attenuation of nocebo hyperalgesia involved expectancies only under operant conditioning.

## 1. Introduction

Nocebo hyperalgesia is the experience of pain increase resulting from an intervention that does not inherently cause such an effect. It is observed in clinical practice,^46^ contributing to patients' discomfort and posing a risk of disrupting therapeutic progress.^30^ Nocebo hyperalgesia is a learning phenomenon acquired through classical conditioning,^5^ operant conditioning,^6^ observational learning,^10,11^ and verbal suggestion.^52^ Given its clinical consequences, it is crucial to explore whether these learning procedures can be also used to attenuate acquired nocebo hyperalgesia.

Prior research has mainly examined counterconditioning (replacing an unwanted response with a desirable one) and extinction (gradual weakening of a conditioned response) in reducing nocebo hyperalgesia.^33,41,53^ While counterconditioning requires repeated pairings of conditioned and unconditioned stimuli and may be clinically impractical, other learning procedures—such as observational learning or operant conditioning—could provide more feasible alternatives. This study examines whether nocebo hyperalgesia induced by pure classical conditioning (without verbal suggestion) can be attenuated by observational learning, counterconditioning, and operant conditioning.

Based on prior findings, we hypothesize that classical conditioning will induce nocebo hyperalgesia,^6,9^ while counterconditioning, verbal modeling, and operant conditioning will reduce it. Building on research on observational learning in placebo effects, this study used verbal modeling—presenting participants with others' pain judgments—to reduce nocebo hyperalgesia, as verbal modeling has been shown to be the most effective in shaping responses to a placebo.^47^ Because social pain-related information can outweigh conditioned cues,^35^ we hypothesize that verbal modeling will attenuate nocebo hyperalgesia more effectively than either counterconditioning or operant conditioning. Evidence that social learning and conditioning involve distinct brain structures^34^ further supports the differentiation between these learning processes. While counterconditioning effectively reduces nocebo hyperalgesia,^41,53^ evidence for operant conditioning remains limited. We hypothesize that counterconditioning, by providing direct experience of reduced pain with placebo, will be more effective than operant conditioning, which shapes placebo effects through external contingencies. Finally, while expectancies are known to mediate placebo and nocebo effects induced by learning procedures,^25,39^ we hypothesize that expectancies will mediate both the induction and reduction of nocebo hyperalgesia.

We also aimed to test whether stress, operationalized here as participants' momentary subjective arousal,^36^ correlates with the nocebo effect. Elevated stress is common in pain patients and linked to increased pain.^3,56^ Although stress influences placebo effects,^47,50^ its role in nocebo attenuation is unknown. We included stress ratings to explore whether momentary stress is linked to attenuation processes. In addition, we explored correlations between nocebo hyperalgesia (during both induction and attenuation) and personality traits, including anxiety, fear of pain, and sensitivity to punishment and reward.

We examined participants' response times to pain applied with and without a placebo, as faster responses may indicate greater confidence in pain assessments.^27^ We also measured electrodermal activity (skin conductance) and electrocardiographic activity (heart rate), which tend to decrease in placebo conditions^17,39^ and increase in nocebo conditions.^23^ These behavioral and autonomic markers were collected to assess whether they covary with responses to a placebo.

## 2. Materials and methods

### 2.1. Study design

Healthy volunteers were randomly allocated to 2 control groups and 3 experimental groups: verbal modeling group (VM), counterconditioning group (CC), operant conditioning group (OC), using computer-generated randomization by sex, ensuring an equal distribution across all 5 groups (1:1:1:1:1). During the induction phase, participants in the experimental groups underwent pure classical conditioning (i.e., not combined with verbal suggestion) to elicit nocebo hyperalgesia. Then, during the attenuation phase, 3 different learning procedures, i.e., verbal modeling, counterconditioning, and operant conditioning, were implemented in the experimental groups to reduce the previously induced nocebo effect. Participants in the control groups underwent sham conditioning during the induction phase. During the attenuation phase, sham conditioning was continued in the sham conditioning control group (SC CTR), while the no-manipulation control group (NM CTR) was not subjected to pain stimuli. The effects induced by the implemented manipulations were measured during 2 testing phases, which occurred after the induction and attenuation phases.

The study protocol received approval from the Research Ethics Committee at the Institute of Psychology, Jagiellonian University, Kraków, Poland, under Reference Number KE/11_2022. In addition, the protocol was preregistered on the Open Science Framework (https://osf.io/x3f6g).

**Figure 1.**
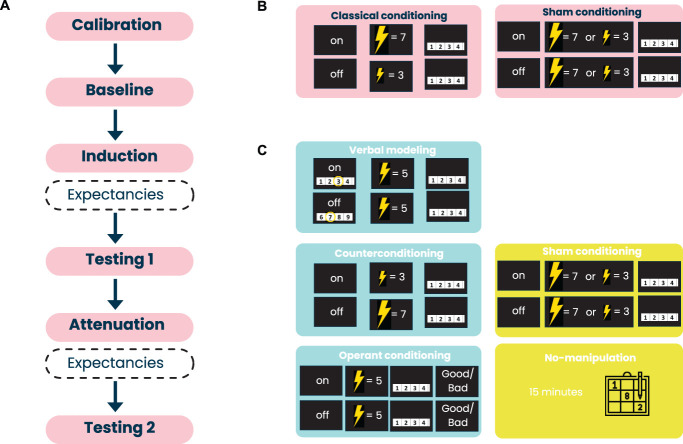
Study design. (A) The experiment consisted of the following phases: calibration, baseline, induction, testing 1, attenuation, and testing 2. Expectancies were measured after the induction phase and after the attenuation phase. (B) In the induction phase, participants in the experimental groups underwent classical conditioning: high-intensity pain stimuli were applied with a placebo, while low-intensity pain stimuli were applied without a placebo. Participants in the control groups underwent sham conditioning: pain stimuli were paired with a placebo in a noncontingent manner. (C) In the attenuation phase, participants in the experimental groups underwent various interventions: (1) verbal modeling (VM)—pain stimuli of moderate intensity were applied with and without a placebo, preceded by the presentation of Numerical Rating Scales demonstrating that other participants allegedly experienced less pain when a placebo was used; (2) counterconditioning (CC)—high-intensity pain stimuli were applied without a placebo, while low-intensity pain stimuli were applied with a placebo; (3) operant conditioning (OC)—moderate-intensity pain stimuli were applied with and without a placebo, and participants were rewarded for reporting lower pain levels and punished for reporting higher pain levels after receiving the placebo. Participants in the control groups underwent sham conditioning (sham conditioning control group; SC CTR) or had a 15-minute break, during which they were asked to solve a Sudoku puzzle (no-manipulation control group; NM CTR). Note: 'on' - activation of placebo device; 'off' - deactivation of placebo device.

The study design is illustrated in Figure 1.

### 2.2. Sample size

Analyses regarding the effectiveness of learning processes in reducing nocebo hyperalgesia were conducted on participants who developed the nocebo response (nocebo responders) in the induction phase; therefore, 2 separate power analyses were performed.

The sample size was established based on the results from a previous similar study on nocebo hyperalgesia attenuation.^54^ Calculation was performed by G*Power^28^ for analysis of variance (ANOVA). A sample size estimate was calculated assuming power (1 − β) = 0.80, probability level α = 0.05, and small effect sizes (ES = 0.25). A sample of at least 42 participants in each group is required to detect a significant difference between groups during the induction phase.

The required sample size for the primary analysis of nocebo responders was calculated based on a previous similar nocebo hyperalgesia attenuation study,^41^ using G*Power for ANOVA analysis.^28^ A sample size estimate was calculated assuming power (1 − β) = 0.80, probability level α = 0.05, and large effect sizes (ES = 0.67). A sample of at least 12 nocebo responders in each group is required to detect a significant difference between the experimental groups during the attenuation phase.

### 2.3. Participants

A total of 168 volunteers, including 98 women (58.33%) (aged between 18 and 50 years), participated in the study. As the study was conducted in Poland, a country with a relatively ethnically homogeneous population and a lack of racial diversity, we did not include a specific question about racial identity in our recruitment survey.

Participants were recruited by posters and advertisement websites. All participants signed a written informed consent to participate in the study and received a financial reward (50 Polish zloty; ∼12 EUR) for their participation.

Before inclusion in the study, all the volunteers underwent online screening. Volunteers who met one or more of the following criteria were excluded from the study: (1) younger than 18 years or older than 50 years; (2) previous participation in a pain study; (3) being a student or graduate of psychology, physiotherapy, or medicine; (4) pain complaints lasting for the past 3 months; (5) taking painkillers or substances containing caffeine and nicotine within 3 hours before the study; (6) use of alcohol within 24 hours before the experiment, or psychoactive drugs, including cannabis, within 72 hours before the study; (7) self-reported current or past diagnosis of a neurological, cardiovascular, metabolic, musculoskeletal, or mental health disorder (assessed based on a Yes/No answer in the recruitment form; any “Yes” answer resulted in exclusion).

### 2.4. Apparatus and materials

#### 2.4.1. Pain stimuli

A Constant Current High Voltage Stimulator (Digitimer, Welwyn Garden City, United Kingdom, Model DS7AH) was used to generate electrocutaneous pain stimuli. The stimulus comprised a series of 3 pulses of 100 μs duration, with an interpulse interval of 200 μs. The stimuli were delivered to the participant's nondominant forearm using a stainless-steel electrode with a diameter of 8 mm and a spacing of 30 mm. The intensity of the electrocutaneous stimuli was determined individually for each participant based on calibration data (see below “Calibration”).

#### 2.4.2. Placebo

An inactive transcutaneous electrical nerve stimulator (TENS) attached to the participant's forearm functioned as a placebo device. Composed of 2 electrodes attached to a stimulus isolator (model FE180, ADInstruments, Dunedin, New Zealand), it was secured with medical tape to the participant's left dorsal forearm.

The alleged activation or deactivation of the placebo device was indicated by displaying a TENS device icon or a crossed-out TENS device icon, respectively, on the computer screen placed in front of the participant.

### 2.5. Measures

#### 2.5.1. Pain intensity and pain expectancy

Pain intensity and pain expectancy were rated on an 11-point Numerical Rating Scale (NRS), ranging from 0 = “no pain” to 10 = “the most pain tolerable.” Participants were asked “How intense was the pain you experienced?” to measure pain intensity, and “How intense pain do you expect?” to measure pain expectancy. The intensity of pain was measured after the application of a pain stimulus. Expectancies were measured twice: after the induction phase and after the attenuation phase.

#### 2.5.2. Response time

Response time (RT) was measured and defined as the time between the presentation of an NRS and the participant's response, entered on the computer keyboard. Response time was measured during testing phases 1 and 2.

#### 2.5.3. Stress

Subjective stress was assessed on an 11-point NRS with the following anchors: 0 = “calm” to 10 = “nervous.” The stress was measured before each phase of the experiment.

#### 2.5.4. Physiological data

The MP-160 hardware system and Acqknowledge v.5.0.2 software were used to collect electrocardiography (ECG) and electrodermal activity (EDA) data during the experiment (BIOPAC systems, Inc., Goleta, CA). To prepare participants for ECG, their skin was abraded with the Nuprep scrub (Weaver and Company, Aurora, CO). Three disposable Ag/AgCl hydrogel (4% chloride salt) electrodes (Ø 11 mm) were connected to the leads and positioned on the participant's left and right lower ribs, as well as the right side of the sternum. To measure the ECG signals, an ECG100D smart amplifier was used (gain x2000, high-pass filter of 1 Hz, low-pass filter of 35 Hz). For EDA, isotonic gel (0.5% chloride salt saline) was used to fill 2 disposable Ag/AgCl electrodes (gel cavity Ø 16 mm), which were placed on the medial phalanges of the index and middle fingers of the nondominant hand. To measure skin conductance level (SCL) and responses (SCR) to the electrical stimuli, the EDA100D smart amplifier was used (low-pass filter of 3 Hz, IIR high-pass 0.5 Hz cut-off, Q = 0.707).

#### 2.5.5. Psychological traits

The scale for cognitive anxiety from the State-Trait Inventory for Cognitive and Somatic Anxiety (STICSA)^45^ was used to measure anxiety. The dispositional tendency to respond with fear to pain was measured by The Fear of Pain Questionnaire (FPQ-III)^38^; Polish adaptation,^37^ which consists of 3 subscales: Severe pain, Minor pain, and Medical pain. Sensitivity to punishment and reward was assessed by a short version of the Sensitivity to Punishment and Sensitivity to Reward Questionnaire (BIS/BAS scales)^21^; Polish adaptation^42^ with 2 subscales: Sensitivity to punishment and Sensitivity to reward.

### 2.6. Procedure

Participants were informed that the purpose of this study was to measure and compare people's objective and subjective responses to pain induced by electrical stimulation. Participants who qualified for the study were asked to fill out an online survey consisting of a few questionnaires measuring psychological traits (for details, see “*Psychological traits*”) before coming to the laboratory. The experimental session consisted of 6 phases: calibration, baseline, induction, testing 1, attenuation, and testing 2 (Fig. 1).

The number of the received pain stimuli varied between participants due to the ascending calibration series. Besides that, all participants in the experimental groups and one control group received the same number of pain stimuli (n = 168), while the other control group had a no-manipulation break during the attenuation phase and therefore received fewer stimuli (n = 120).

#### 2.6.1. Calibration

Calibration was conducted to determine 3 electrocutaneous stimuli that elicited pain at intensities rated by the participant as “3,” “5,” and “7” on NRS. To determine these stimuli, an approximate polynomial function was used: STIM(N) = aN^2^² + bN + c. In this equation, N represents the NRS rating and STIM(N) indicates the intensity of the stimuli corresponding to that specific NRS rating.

The calibration consisted of 2 parts. First, an ascending series of stimuli was delivered in steps of 1 mA (the interstimulus interval was 5 seconds), starting from 0 mA. Participants rated the intensity of pain elicited by each stimulus on the NRS. The stimulation increased until it elicited pain that participants assessed as 9 or 10 on the NRS, resulting in variation in the number of stimuli received across individuals. Second, the resulting function was used to determine stimuli whose intensities correspond to 2, 3, 4, 5, 6, 7, and 8 points on the NRS. Two identical sequences of pseudorandom stimuli were applied: “3-6-5-4-8-7-5-3-7-2-3-7-5-4-8-6-2.” Participants rated the intensity of pain induced by each stimulus on the NRS. The NRS ratings obtained during the secondary phase of calibration were subsequently used to obtain the final coefficients of the STIM(N) function. Calibration involved 7 target NRS levels (2-8), with a total of 34 stimuli administered. STIM(N) function was fitted to the mean responses across these levels, resulting in a mean R^2^ = 0.996 (range: 0.722-1.000).

#### 2.6.2. Baseline

Baseline was designed to assess the effectiveness of the calibration and to determine whether participants respond differently to target stimuli. A total of 6 stimuli of 3 predetermined intensities were applied in a pseudorandom sequence (either “3-7-3-5-7-5” or “7-3-7-5-3-5”). The stimuli sequences were counterbalanced between participants.

#### 2.6.3. Induction

The purpose of this phase was to induce nocebo hyperalgesia in the experimental groups through classical conditioning. First, the placebo device (TENS) was attached to the participant's nondominant forearm. Participants were informed that this device would be activated periodically, but they were not informed about the potential effects it might cause. Immediately after the device was placed on the forearm, it was activated and operated until the participant felt mild tingling sensations. The purpose of this procedure was to convince the participants that TENS is an active device that may potentially affect the body. Then, the device was switched off and remained inactive during the entire study.

Next, the participants in the experimental groups received 16 pain stimuli applied in 3 blocks, with 1-minute breaks in between. In each block, half of the stimuli were administered with a placebo (an icon indicating alleged activation of TENS was displayed), while the other half were administered without a placebo (an icon indicating deactivation of TENS was shown). During the placebo trials, stimuli at intensity corresponding to “7” points on the NRS were applied, while in the nonplacebo trials, stimuli at intensity corresponding to “3” points on the NRS were delivered. The participants in the control groups underwent sham conditioning, i.e., they were subjected to the same procedure with one exception: the alleged activation of the placebo device was not related in any way to the intensity of the applied stimuli.

#### 2.6.4. Testing 1

A total of 16 pain stimuli at 3 predetermined intensities were applied. To verify whether nocebo hyperalgesia was induced, 8 stimuli at moderate intensity (“5”) were applied with or without a placebo. To prevent the extinction of conditioned nocebo hyperalgesia during this phase, booster trials were implemented: stimuli at high intensity (“7”) were applied in 4 placebo trials, and stimuli at low intensity (“3”) were administered in 4 nonplacebo trials. Stimuli were applied in the order “5,5,3,7,5,5,7,3,5,5,7,3,5,5,3,7.”

#### 2.6.5. Attenuation

The purpose of this phase was to reduce nocebo hyperalgesia induced by classical conditioning. In the experimental groups and one control group (SC CTR), a total of 16 pain stimuli were applied in 3 blocks, with 1-minute breaks in between. Half of the stimuli in each block were applied with a placebo, and the other half were applied without a placebo.

##### 2.6.5.1. Operant conditioning group

The participants were informed that in this phase of the experiment, they would receive feedback on whether their subjective pain experiences were consistent with their physiological responses to pain stimuli. The feedback, displayed on the computer screen, functioned either as a reinforcer (“Good!”) or as a punisher (“Wrong! Too low” or “Wrong! Too high”). During both placebo and nonplacebo trials, stimuli at moderate intensity were applied (“5”). Participants rated the intensity of pain induced by each stimulus and were rewarded for experiencing low pain level (≤“4”) in placebo trials and for experiencing high pain level (≥“5”) in nonplacebo trials. Participants were also punished for experiencing high pain level (≥“5”) in placebo trials, and for experiencing low pain level (≤4 on the NRS) in nonplacebo trials.

In our study, the presence of the placebo served as the environmental condition (antecedent) in which pain experiences, as expressed through verbal pain reports (behavior), were modified by immediate feedback from the experimenter (consequence).

##### 2.6.5.2. Verbal modeling group

Before each stimulus, the participants were presented with a pain rating displayed on the NRS and were informed that this rating represents the average of the ratings provided by other participants who had been exposed to the same stimulus. During both placebo and nonplacebo trials, stimuli at moderate intensity were applied (“5”). The pain ratings presented in the placebo trials indicated that other participants allegedly experienced low-level pain (2-4 on the NRS), while the ratings presented in nonplacebo trials suggested that they allegedly experienced high-level pain (6-8 on the NRS).

##### 2.6.5.3. Counterconditioning group

During the placebo trials, stimuli at lower intensity were applied (“3”), whereas stimuli at higher intensity (“7”) were delivered in the nonplacebo trials.

##### 2.6.5.4. Control groups

In 1 of the 2 control groups (SC CTR), sham conditioning was continued, while in the other control group (NM CTR), participants were not subjected to pain stimuli. To fill the interval between testing 1 and testing 2, participants in the NM CTR group solved Sudoku puzzles.

#### 2.6.6. Testing 2

To verify whether nocebo hyperalgesia was effectively attenuated, 16 stimuli at moderate intensity (“5”) were applied with or without a placebo.

#### 2.6.7. Closing questions

When the experiment was completed, participants were asked questions to determine (1) whether they had figured out the aim of the study; (2) whether the TENS device had influenced their experience of pain; (3) if so, in what way the TENS device had influenced their pain perception; (4) whether they observed any change in the functioning of the TENS device throughout the experiment; (5) in the operant conditioning and verbal modeling groups, participants were also asked how much they tried to adjust their pain ratings to, respectively, the received feedback or information about others' pain ratings.

### 2.7. Statistical analyses

As a first step, a sensitivity analysis was conducted to assess the impact of outliers (defined as values exceeding 3 times the interquartile range [IQR]) and participants who correctly guessed the true aim of the study on the main outcomes of interest—namely, nocebo hyperalgesia induction and the effectiveness of attenuation procedures. Based on the results of these analyses, decisions were made regarding the inclusion or exclusion of extreme cases and potentially biased participants in the final analyses.

As preliminary analyses, we examined whether the groups differed in baseline pain and stress ratings, sociodemographic characteristics, and personality traits. Group differences in continuous variables were tested with one-way analyses of variance (ANOVA), while differences in categorical variables were assessed with χ^2^ tests.

To test whether classical conditioning will effectively induce nocebo hyperalgesia, the independent samples Welch *t* test was conducted to account for heterogeneity of variance (a 1-way ANOVA was initially planned in the preregistration; however, due to heterogeneity of variance, we adjusted the analysis and performed Welch *t* test instead). The analysis focused on the difference in mean pain ratings between placebo and nonplacebo trials during testing 1 (excluding booster trials), with group (merged experimental groups vs merged control groups) as the between-subjects factor. A 2 × 2 mixed-model ANOVA with group (merged experimental groups vs merged control groups) as a between-subjects factor and trial (placebo vs nonplacebo) as a within-subjects factor was performed for physiological measures (heart rate [HR], SCL, SCR) and RT to explore whether classical conditioning influenced these parameters. Heart rate, skin conductance level, and skin conductance responses were log-transformed (_lg10_) to address non-normality.

To test the hypotheses regarding effectiveness of counterconditioning, verbal modeling, and operant conditioning in attenuating nocebo hyperalgesia, a 3 × 2 mixed-model ANOVA followed by pairwise comparisons (with Bonferroni correction) was conducted on data from nocebo responders, who were defined as participants from experimental groups with a positive difference in mean pain ratings between placebo and nonplacebo trials during testing 1. Group (VM, CC, and OC) was included as a between-subjects factor, and nocebo attenuation was included as a within-subjects factor in the analysis. Nocebo attenuation was operationalized as the difference in mean pain ratings between the placebo and nonplacebo trials during testing 1 (excluding booster trials) and the first 4 placebo and nonplacebo trials of testing 2. The same number of trials (4 placebo and 4 nonplacebo) from testing 2 was used for the mean calculations to ensure consistency. An additional 3 × 2 × 2 mixed-model ANOVA with group as a between-subjects factor and phase (testing 1 vs the first 4 trials of testing 2) and trial (placebo vs nonplacebo) as within-subjects factors was performed for mean HR_lg10_, SCL_lg10_, SCR_lg10_, and RT to explore the effect of different attenuation procedures on physiological outcomes. Bonferroni-corrected pairwise comparisons were used to evaluate significant effects.

Finally, to examine attenuation effects in comparison with control conditions, a 5 × 2 explanatory mixed-model ANOVA was performed on nocebo responders. The between-subjects factor included group (VM, CC, OC, SC CTR, and NM CTR), while the within-subjects factor was nocebo attenuation. The analysis was followed by pairwise comparisons.

To assess the stability of nocebo attenuation effects over time, a 3 × 2 mixed-model ANOVA was conducted (exploratory analysis). The between-subjects factor was group (VM, CC, and OC), and the within-subjects factor was the stability of nocebo attenuation effects (the difference in mean pain ratings between the first and the last placebo vs nonplacebo trials in testing 2). As before, analyses were conducted on data from nocebo responders, and Bonferroni-corrected pairwise comparisons followed the ANOVA.

Mediation analyses were conducted to test the hypothesis whether differences in expectancy ratings mediated nocebo hyperalgesia and its attenuation. In these analyses, the group was treated as the independent variable, with dummy coding used to compare the experimental groups against the merged control groups, which served as the reference category. In the analysis of nocebo hyperalgesia, the independent variable was group (merged experimental groups vs merged control groups), dependent variable was the difference in pain ratings between placebo and nonplacebo trials during testing 1 (excluding booster trials), with the corresponding difference in expectancy ratings included as the mediator. In the analysis of nocebo attenuation, the dependent variable was the change in mean pain ratings for placebo trials between testing 2 (first 4 trials) and testing 1, with the difference in expectancy ratings between the attenuation and induction phases serving as the mediator. Two separate analyses of nocebo attenuation were conducted: one comparing the combined experimental groups with the combined control groups (primary analysis), and another comparing each experimental group individually with the combined control groups, to account for the possibility of distinct mechanisms underlying the different attenuation methods (exploratory analysis). These analyses were conducted on nocebo responders identified during testing 1 phase. To evaluate the significance of indirect effects, bias-corrected bootstrap 95% confidence intervals were calculated using 5000 resamples.

Correlation analyses (Pearson) were performed to test the hypotheses regarding the relationship between subjective stress and the magnitude of induced nocebo hyperalgesia, as well as the effects of nocebo attenuation. Nocebo hyperalgesia and nocebo attenuation were operationalized in the same manner as in the mediation analyses. Additional correlation analyses were conducted to explore associations between individual psychological traits (e.g., anxiety, fear of pain, and sensitivity to punishment and reward) and magnitude of nocebo hyperalgesia and effects of nocebo attenuation.

A 5 × 5 mixed-model ANOVA with group as the between-subjects factor (VM, CC, OC, SC CTR, NM CTR) and phase (baseline, after baseline, after induction, after attenuation, after testing 2) as a within-subjects factor, followed by the pairwise comparisons, was conducted to explore whether the level of stress changed during the experiment. In addition, a Mann–Whitney *U* test was used to compare responses to the control questions, each measured on an ordinal scale. Because not all questions were administered to every group, the following comparisons were made: (1) merged control vs merged experimental groups regarding whether the TENS device influenced pain ratings; (2) merged control vs CC groups regarding whether participants noticed significant changes in the TENS device's functioning; and (3) OC vs VM groups regarding the extent to which the provided information influenced subjective pain ratings and whether participants consciously tried to adjust their ratings to align with that information. These exploratory tests helped to contextualize the primary findings.

All statistical analyses were performed using the PS IMAGO PRO software (version 9), which uses IBM SPSS Statistics as its analytical engine (version 29, SPSS Inc, Chicago, IL). One-sided significance tests were applied to predefined comparisons with directional hypotheses. For ANOVA post hoc tests, one-sided tests were conducted only when the overall main or interaction effects were significant and the specific comparison aligned with the predefined directional hypotheses. When a one-sided test was used, it is explicitly marked as “*P*_one-sided_.” Mediation analyses were conducted using the Process Macro for SPSS version 4.1,^31^ using model 4 for simple mediation (when issues with homoscedasticity were detected, the HC3 standard error estimator was used). The significance level for all tests was set at *α* = 0.05. When the sphericity assumption was violated in the repeated-measures ANOVA, the Greenhouse–Geisser correction was applied to the degrees of freedom. Cases with missing data were excluded using listwise deletion. The PhysioData Toolbox 0.6.3^49^ was used for visual inspection of the physiological data (HR, SCL, SCR) and calculations of the data end points.

## 3. Results

### 3.1. Sensitivity analysis

The sensitivity analysis was conducted in 2 phases. First, we assessed the impact of outliers on the main results of interest concerning nocebo hyperalgesia induction and the effectiveness of attenuation procedures. Second, we evaluated whether excluding participants who correctly guessed the true aim of the study would alter these findings. To explore the potential effects of outliers and participants' knowledge of the study's purpose, we performed all analyses testing the study's hypotheses twice: once including and once excluding these cases. Outliers were identified using box plots, applying the criterion of values greater than 3 IQR below the first quartile and above the third quartile. This process revealed 3 outliers. Sensitivity analyses showed that excluding these outliers changed the results for nocebo induction (see Supplementary Table A, http://links.lww.com/PAIN/C459) but had no impact on the findings related to nocebo attenuation (see Supplementary Table C, http://links.lww.com/PAIN/C459). Given their influence on the induction phase and to ensure consistency across all analyses, these outliers were excluded from the final analyses, resulting in the final sample of 165 participants. By contrast, excluding participants who had correctly guessed the true aim of the study (N = 18) did not affect the results. Therefore, these participants were retained in the analyses.

### 3.2. Preliminary analyses

Before testing the hypotheses, we assessed whether the groups differed in baseline pain and stress ratings, sociodemographic factors, and personality characteristics. No significant differences were found (all *P* > 0.24), suggesting that the groups were comparable. Details are summarized in Table 1.

**Table 1:** Descriptive statistics and differences between groups in baseline characteristics.

Variable	Mean (SD)					*P* *
	VM (N = 43)	CC (N = 40)	OC (N = 41)	SC CTR (N = 21)	NM CTR (N = 20)	
Age	23.28 (6.16)	23.92 (7.51)	23.39 (5.07)	23.79 (8.44)	26.55 (6.89)	0.448
BMI	22.24 (5.06)	21.44 (3.98)	22.12 (5.66)	21.54 (4.70)	22.92 (5.26)	0.841
Baseline pain ratings						
Ratings for low pain stimuli	2.86 (1.24)	2.63 (1.64)	3.01 (1.51)	2.76 (1.04)	2.58 (1.52)	0.723
Ratings for moderate pain stimuli	4.66 (1.34)	4.21 (1.48)	4.82 (1.44)	4.57 (0.87)	4.75 (1.09)	0.306
Ratings for high pain stimuli	5.24 (1.47)	5.10 (1.84)	5.43 (1.78)	5.43 (1.55)	5.93 (1.05)	0.437
Baseline stress rating	1.79 (1.66)	1.53 (1.65)	1.41 (1.47)	1.48 (1.63)	1.65 (1.76)	0.856
FPQ-III						
Severe pain	29.79 (7.70)	28.11 (8.05)	27.51 (7.68)	28.28 (8.35)	27.95 (8.56)	0.764
Minor pain	17.90 (5.32)	17.47 (5.05)	17.87 (5.65)	18.61 (6.42)	17.10 (4.82)	0.925
Medical pain	23.64 (7.19)	22.16 (6.66)	21.73 (6.47)	23.83 (8.11)	21.45 (7.50)	0.609
SPSRQ						
Sensitivity to rewards	4.80 (2.21)	5.24 (2.45)	5.24 (2.09)	5.28 (2.49)	4.74 (2.05)	0.814
Sensitivity to punishments	6.12 (3.55)	4.46 (2.97)	5.87 (3.54)	5.56 (3.68)	5.16 (2.97)	0.240
STICSA	20.38 (5.74)	18.84 (4.85)	20.03 (6.19)	19.78 (4.24)	20.00 (6.52)	0.800
No. of participants in each subgroup						
Sex (men/women)	18/25	16/24	18/23	9/12	8/12	0.997
Handedness (right-handed/left-handed)	41/2	38/2	39/2	18/3	20/0	0.351
Education (primary/secondary/vocational/higher)	2/29/1/8	3/23/2/8	0/24/0/14	1/13/0/5	1/11/0/8	0.530
Employment status (student/employed/unemployed)	30/6/4	25/6/5	30/6/2	15/2/2	12/5/3	0.860

* ANOVA was calculated for continuous variables; χ^2^ test was used for nominal data; none of the participants reported ambidexterity.

BMI, body mass index; CC, counterconditioning; FPQ-III, Fear of Pain Questionnaire-III; NM CTR, no manipulation control group; OC, operant conditioning; SC CTR, Sham conditioning control group; SPSRQ, Sensitivity to Punishment and Sensitivity to Reward Questionnaire; STICSA, State-Trait Inventory for Cognitive and Somatic Anxiety; VM, verbal modeling.

### 3.3. Induction of nocebo hyperalgesia

#### 3.3.1. Pain ratings

The Welch independent samples *t* test revealed a statistically significant difference between the merged experimental groups and the merged control groups regarding the difference in mean pain ratings between placebo and nonplacebo trials during the testing 1 phase, t(102.60) = −1.82, *P*_*one-sided*_ = 0.036, d = −0.30, M_diff_ = −0.19, indicating that nocebo hyperalgesia was successfully induced in the experimental groups, albeit with a small effect size. The means and standard deviations of pain ratings across different groups, trials, and phases of the experiment are summarized in the Supplementary material (Supplementary Table B1, http://links.lww.com/PAIN/C459).

#### 3.3.2. Physiological measures and response time

Additional mixed 2 (merged experimental groups vs merged control groups) × 2 (placebo vs nonplacebo trials) ANOVAs were conducted on physiological measures and RT to determine whether the effects observed in pain ratings were reflected in these measures. For HR_lg10_, neither the main effect of trial, *F*(1,161) = 1.71, *P* = 0.193, η^2^G = 0.00, nor the main effect of group, *F*(1,161) = 2.81, *P* = 0.10, η^2^G = 0.02, nor the group × trial interaction, *F*(1,161) = 1.60, *P* = 0.208, η^2^G = 0.00, was significant. For SCL_lg10_, a significant but very small main effect of trial was observed, *F*(1,161) = 19.12, *P* < 0.001, η^2^G = 0.00, with higher mean SCL_lg10_ registered during placebo trials compared with nonplacebo trials. The main effect of group, *F*(1,161) = 1.89, *P* = 0.171, η^2^G = 0.01, and the group × trial interaction, *F*(1,161) = 0.50, *P* = 0.480, η^2^G = 0.00, were not significant. No significant effects were found for SCR_lg10_ (main effect of group: *F*(1,125) = 1.79, *P* = 0.185, η^2^G = 0.01; main effect of trial: *F*(1,125) = 1.12, *P* = 0.292, η^2^G = 0.00; group × trial interaction: *F*(1,125) = 0.31, *P* = 0.578, η^2^G = 0.00).

A significant main effect of trial was found for RT, *F*(1,161) = 6.58, *P* = 0.011, η^2^G = 0.01, with RTs being lower in nonplacebo trials compared with placebo trials. The main effect of group, *F*(1,161) = 3.00, *P* = 0.085, η^2^G = 0.02, and the group × trial interaction, *F*(1,161) = 0.09, *P* = 0.768, η^2^G = 0.00, were not significant.

### 3.4. Attenuation of nocebo hyperalgesia

#### 3.4.1. Pain ratings

**Figure 2.**
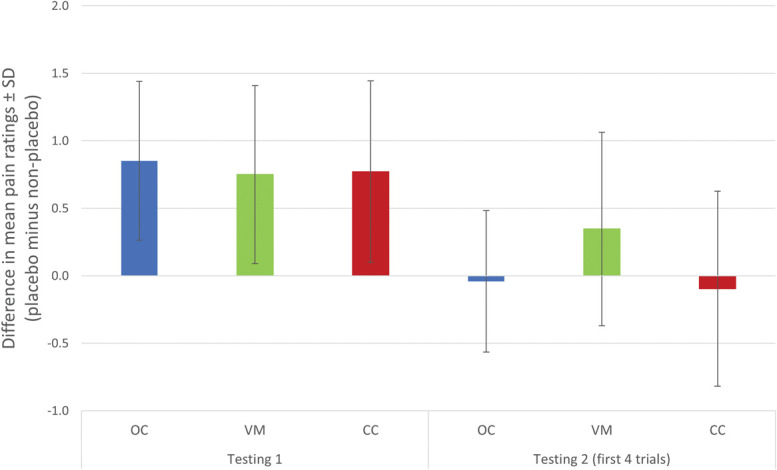
Difference in mean pain ratings between placebo and nonplacebo trials for the experimental groups during testing 1 and the first 4 trials of testing 2. Note: N = 63; analysis conducted on nocebo responders. CC, counterconditioning group; OC, operant conditioning group; VM, verbal modeling group.

The 3 (group: VM vs CC vs OC) × 2 (nocebo attenuation) mixed-model ANOVA revealed a statistically significant effect of the experiment's phase (testing 1 vs the first 4 trials in testing 2) on the difference in mean pain ratings between placebo and nonplacebo trials, *F*(1,60) = 35.40, *P* < 0.001, η^2^G = 0.24. Across all groups, the difference in mean pain ratings between placebo and nonplacebo trials decreased from testing 1 to testing 2 (see Fig. 2 and Supplementary Table B2, http://links.lww.com/PAIN/C459). The effect of group (VM vs CC vs OC) was not significant, *F*(2, 60) = 1.28, *P* = 0.285, η^2^G = 0.02, nor was the interaction between phase and group, *F*(2, 60) = 1.88, *P* = 0.161, η^2^G = 0.03.

#### 3.4.2. Physiological measures and response time

The 3 (group: VM vs CC vs OC) × 2 (phase: testing 1 vs the first 4 trials of testing 2) × 2 (trial: placebo vs nonplacebo) mixed-model ANOVA revealed a significant interaction effect of phase and group for HR_lg10_ was observed, *F*(2,59) = 4.78, *P* = 0.012, η^2^G = 0.01. Pairwise comparisons revealed that mean HR_lg10_ in the VM group decreased significantly from testing 1 to the first 4 trials of testing 2 (*P* = 0.043, *d* = 0.14), whereas mean HR_lg10_ in the CC group increased significantly in the same period (*P* = 0.029, *d* = 0.17). By contrast, no significant difference in mean HR_lg10_ was found between phases in the OC group (*P* = 0.592, *d* = 0.05). The main effects of phase, group, and trial, as well as trial × group, phase × trial, and phase × trial × group interactions were not significant (all *P* > 0.35).

For SCL_lg10_, significant main effects were found for group, *F*(2,59) = 3.31, *P* = 0.043, η^2^G = 0.10; phase *F*(1,59) = 16.48, *P* < 0.001, η^2^G = 0.00; and trial, *F*(1,59) = 6.04, *P* = 0.017, η^2^G = 0.00. In addition, the group × phase × trial interaction was significant, *F*(2,59) = 4.75, *P* = 0.012, η^2^G = 0.00. The phase × group, trial × group, and phase × trial interactions were not significant (all *P* > 0.09).

**Figure 3.**
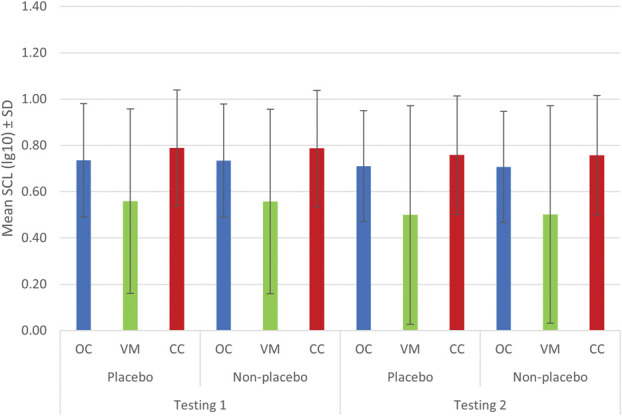
Mean SCL_lg10_ (log transformed) for the placebo and nonplacebo trials in experimental groups during testing 1 and the first 4 trials of testing 2. Note: *P < 0.05, ***P < 0.001; N = 62; analysis conducted on nocebo responders. CC, counterconditioning group; OC, operant conditioning group; VM, verbal modeling group.

Pairwise comparisons exploring the 3-way interaction indicated that in the VM group, for both placebo (*P* < 0.001, *d* = 0.18) and nonplacebo (*P* < 0.001, *d* = 0.17) trials, mean SCL_lg10_ decreased significantly from testing 1 to the beginning of testing 2 (Fig. 3). However, in the OC and CC groups, no significant differences in mean SCL_lg10_ between phases were found for either placebo or nonplacebo trials (all *P* > 0.06).

Further analyses showed that SCL_lg10_ during testing 1 was higher in placebo trials than in nonplacebo trials within both the VM (*P* = 0.026, *d* = −0.01) and the CC (*P* = 0.043, *d* = −0.01) groups. However, this difference was not significant in testing 2 (all *P* > 0.17). By contrast, the OC group exhibited the opposite pattern: no significant difference between placebo and nonplacebo trials during testing 1 (*P* = 0.069, *d* = −0.00) but a significant difference during testing 2, with higher SCL_lg10_ in placebo trials (*P* = 0.011, *d* = −0.01).

A significant main effect of phase was observed for SCR_lg10_, *F*(1,36) = 17.51, *P* < 0.001, η^2^G = 0.04, with mean SCR_lg10_ being higher in testing 1 compared with the beginning of testing 2. In addition, a significant group × trial interaction effect was found, *F*(2,36) = 4.93, *P* = 0.013, η^2^G = 0.02. Pairwise comparisons revealed that in the OC group, mean SCR_lg10_ was significantly lower for placebo trials compared with nonplacebo trials (*P* = 0.039, *d* = 0.36). By contrast, the VM group showed the opposite pattern, with mean SCR_lg10_ significantly higher for placebo trials than for nonplacebo trials (*P* = 0.031, *d* = −0.40). In the CC group, the difference between placebo and nonplacebo trials was not significant (*P* = 0.564, *d* = −0.09). The main effects of trial and group, as well as phase × group, phase × trial and phase × trial × group interactions, were not significant (all *P* > 0.11).

For RT, significant main effects were observed for phase, *F*(1,61) = 26.63, *P* < 0.001, η^2^G = 0.06, and trial, *F*(1,61) = 9.83, *P* = 0.003, η^2^G = 0.01, along with a significant phase × trial interaction, *F*(1,61) = 4.22, *P* = 0.044, η^2^G = 0.01. Pairwise comparisons examining the interaction effect indicated that the difference between trials was not significant during testing 1 (*P* = 0.744, *d* = −0.03). However, in testing 2, the difference became significant (*P* < 0.001, *d* = −0.35), with response times being shorter for nonplacebo trials compared with placebo trials. For both types of trials, mean RT was shorter in testing 2 than in testing 1 (*P* = 0.006, *d* = 0.35 for placebo trials and *P* < 0.001, *d* = 0.67 for control trials). Main effect of group, as well as the phase × group, trial × group, and phase × trial × group interactions were not significant (all *P* > 0.14).

#### 3.4.3. Additional analysis

**Figure 4.**
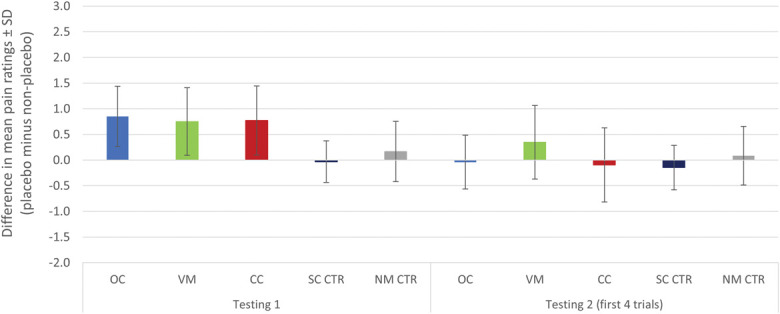
Difference in mean pain ratings between placebo and nonplacebo trials for the 3 experimental and the 2 control groups during testing 1 and the first 4 trials of testing 2. Note: *P < 0.05, **P < 0.01, ***P < 0.001; N = 104; analysis conducted on nocebo responders. CC, counterconditioning group; NM CTR, no manipulation control group; OC, operant conditioning group; SC CTR, sham conditioning control group; VM, verbal modeling group.

An additional 5 (VM vs CC vs OC vs SC CTR vs NM CTR) × 2 (testing 1, first 4 trials in testing 2) mixed-model ANOVA was conducted to further explore the effect of nocebo attenuation. The analysis revealed a statistically significant interaction between group and phase, *F*(4,99) = 4.06, *P* = 0.004, η^2^G = 0.08. Pairwise comparisons revealed significant differences between phases within the experimental groups (all *P* < 0.03), but not within the control groups (all *P* > 0.54). As expected, given that the analysis focused on nocebo responders, all experimental groups exhibited a greater difference between placebo and nonplacebo trials during testing 1 compared with the control groups (all *P* < 0.02). Importantly, these differences disappeared during the first 4 trials of testing 2 (Fig. 4).

### 3.5. Stability of the nocebo attenuation effects

A 3 (group: VM vs CC vs OC) × 2 (phase: the first vs the last placebo vs nonplacebo trials in testing 2) mixed-model ANOVA conducted on pain ratings did not reveal any significant effects. The main effect of group was *F*(2,60) = 2.66, *P* = 0.078, η^2^G = 0.05; the main effect of phase was *F*(1,60) = 0.43, *P* = 0.517, η^2^G = 0.00; and the group × phase interaction was *F*(2,60) = 1.40, *P* = 0.254, η^2^G = 0.02. Therefore, we did not find evidence that the attenuation effect disappeared during testing 2.

### 3.6. Expectancies as mediators of nocebo hyperalgesia

**Figure 5.**
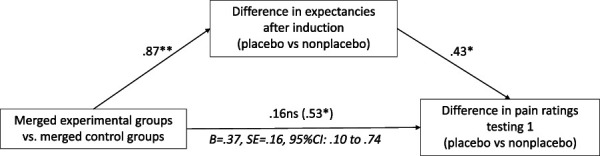
Expectancies as mediators of nocebo hyperalgesia. Note: *P < 0.05, **P < 0.01; N = 57; regression coefficients (ß) are presented; indirect effects are written in italics; total effects are presented in brackets; due to issues with homoscedasticity, the HC3 standard error estimator was used, R^2^ = 0.20, *F*(2,54) = 5.92, *P* = 0.005.

The mediation analysis revealed a significant effect of group (merged experimental vs merged control groups) on expectancies: β = 0.87, *P* = 0.004. After the induction, the merged experimental groups exhibited a greater difference in expectancies between placebo and nonplacebo trials compared with the merged control groups. Furthermore, a larger difference in expectancies between placebo and nonplacebo trials was associated with a greater difference in mean pain ratings for these trials during testing 1: β = 0.43, *P* = 0.011. The total effect of group on the difference in pain ratings between placebo and nonplacebo trials was significant: β = 0.53, *P* = 0.017. However, this effect became nonsignificant after including the mediator: β = 0.16, *P* = 0.479. Moreover, the indirect effect of group through expectancies was significant, β = 0.37, *SE* = 0.16, 95% CI: 0.10 to 0.74, indicating full mediation (Fig. 5).

### 3.7. Expectancies as mediators of nocebo attenuation

The mediation analysis examining the role of expectancies in nocebo attenuation revealed a significant association between the change in pain expectancies from the induction to the attenuation phase (placebo trials) and the change in pain ratings from testing 1 to testing 2 (also placebo trials): β = 0.41, *P* < 0.001. However, the change in expectancies did not significantly differ between the merged experimental and merged control groups, β = −0.31, *P* = 0.164. The indirect effect of group on pain ratings through expectancies change was also not significant, β = −0.13, *SE* = 0.10, 95% CI: −0.33 to 0.06 (see Supplementary Figure A, http://links.lww.com/PAIN/C459 for details).

**Figure 6.**
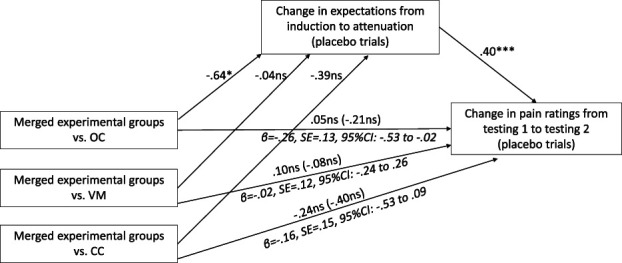
Pain expectancies as a mediator of the nocebo attenuation effect. Note: *P < 0.05, ***P < 0.001; ns, not significant; N = 82; standardized regression coefficients (ß) are presented; indirect effects are written in italics; total effects are presented in brackets; analysis was conducted on nocebo responders; R^2^ = 0.18, *F*(4, 77) = 4.25; *P* < 0.01.

Given that different nocebo attenuation methods may operate through distinct mechanisms, additional mediation analyses were conducted with each experimental group analyzed separately against the merged control groups. Although the direct and total effects of group comparisons were not significant, a significant indirect effect was observed for the OC group: the effect of group on pain ratings was mediated by changes in expectancies (β = −0.26, SE = 0.13, 95% CI: –0.53 to −0.02), indicating an “indirect-only” mediation (see Fig. 6 for details). By contrast, the indirect effects for the VM group (β = −0.02, SE = 0.12, 95% CI:–0.24 to 0.26) and the CC group (β = −0.16, SE = 0.15, 95% CI: −0.53 to 0.09) were not significant.

### 3.8. Relationship between stress, psychological traits, nocebo hyperalgesia, and nocebo attenuation

Correlations between nocebo hyperalgesia, nocebo attenuation, stress ratings, and psychological traits were not statistically significant (all *P* > 0.09, see Table 2 for details).

**Table 2:** Correlations (Pearson r) between nocebo hyperalgesia, nocebo attenuation, and stress experienced in different phases of the experiment and psychological traits.

	Nocebo hyperalgesia	Nocebo attenuation
Stress at baseline	0.073	0.060
Stress after baseline	0.075	0.083
Stress after induction	0.072	0.109
Stress after attenuation	0.067	0.216
Stress after testing 2	0.041	0.178
FPQ-III Severe pain	−0.015	0.023
FPQ-III Minor pain	−0.004	−0.160
FPQ-III Medical pain	0.037	−0.111
SPSRQ Sensitivity to rewards	0.042	−0.109
SPSRQ Sensitivity to punishments	0.156	−0.115
STICSA Cognitive anxiety	0.031	−0.128

Note: N = 113 for correlations with nocebo hyperalgesia; N = 58 for correlations with nocebo attenuation. Nocebo hyperalgesia was operationalized as the difference between placebo and nonplacebo trials in testing 1 within merged experimental groups. Nocebo attenuation was operationalized as the difference between testing 1 and first trials in testing 2 for placebo trials within merged experimental groups (nocebo responders).

FPQ-III, Fear of Pain Questionnaire-III; SPSRQ, Sensitivity to Punishment and Sensitivity to Reward Questionnaire; STISCA, State-Trait Inventory for Cognitive and Somatic Anxiety.

### 3.9. Exploratory analyses

#### 3.9.1. Change in stress ratings

To examine whether stress levels changed throughout the experiment depending on group allocation, a 5 × 5 mixed-model ANOVA was conducted with group as the between-subjects factor (VM, CC, OC, SC CTR, NM CTR), and phase (baseline, after baseline, after induction, after attenuation, after testing 2) as a within-subjects factor. Only the main effect of phase was statistically significant: *F*(2.55, 402.95) = 9.82, *P* < 0.001, η^2^G = 0.02. The effects of group, *F*(4,158) = 0.62, *P* = 0.652, η^2^G = 0.02, and the phase × group interaction, *F*(10.20, 402.95) = 1.20, *P* = 0.290, η^2^G = 0.01, were not significant. Pairwise comparisons revealed that stress levels measured after baseline were significantly lower than in all other phases (all *P* ≤ 0.003).

#### 3.9.2. Closing questions

A Mann–Whitney *U* test revealed no significant difference in participants' belief that the TENS device affected their pain sensations between the merged experimental (N = 124, *Mdn* = 3, range = 1-5) and merged control (N = 41, *Mdn* = 2, range = 1-5) groups, *U* = 2985.50, *P* = 0.087. Similarly, there was no significant difference between the merged control groups (N = 41, *Mdn* = 2, range = 1-5) and the CC group (N = 40, *Mdn* = 3, range = 1-5) regarding perceived changes in the TENS device's functioning, *U* = 1013.00, *P* = 0.061. By contrast, the OC and VM groups differed significantly in their belief that the information provided during the study influenced their pain experience, *U* = 396.00, *P* < 0.001, with the OC group reporting a stronger perceived influence (N = 40, *Mdn* = 3, range = 1-4) than the VM group (N = 43, *Mdn* = 1, range = 0-3). The 2 groups also differed significantly in the extent to which they consciously adjusted their pain intensity ratings according to the provided information, such that the OC group (N = 40, *Mdn* = 3, range = 0-4) reported greater adjustment than the VM group (N = 43, *Mdn* = 1, range = 0-4).

## 4. Discussion

This study is the first to show that verbal modeling, counterconditioning, and operant conditioning effectively reduce nocebo hyperalgesia induced by classical conditioning, with no differences in effectiveness. Expectancies mediated induction and, for operant conditioning, attenuation of nocebo hyperalgesia.

Nocebo hyperalgesia was successfully shaped by classical conditioning, supporting our first hypothesis. Unlike studies that combine conditioning with verbal suggestion,^33,53^ our study demonstrates that conditioning alone can be effective.^7,9^ This highlights the ease with which nocebo hyperalgesia can arise and the need for strategies to prevent or reduce it.

The hypotheses that verbal modeling, counterconditioning, and operant conditioning would reduce nocebo hyperalgesia were confirmed, suggesting that this effect is dynamic and modifiable once established. Unlike previous studies, in which procedures aimed at reversing nocebo hyperalgesia immediately followed the induction procedure,^15^ this study first verified whether nocebo hyperalgesia had been successfully induced. Moreover, unlike earlier work,^14,15,18^ the effectiveness of reversal procedures was assessed only in participants who exhibited a nocebo response.

This study offers new evidence that operant conditioning can modify the nocebo effect. Verbal cues (“good”/“wrong”) acted as effective reinforcers and punishers, and post-experiment questions indicated participants attended to and were influenced by them. Although we did not assess whether partici-pants recognized the contingency between their pain responses and the feedback they received, previous research suggests that such awareness is not necessary for operant learning.^16,43^ While operant learning is known to produce placebo hypoalgesia,^1,19^ our results demonstrate that it can also reduce nocebo hyperalgesia acquired through prior experience, highlighting its broader potential in modulating treatment responses.

Verbal modeling, in which participants were exposed to pain ratings provided by others, also effectively reduced nocebo hyperalgesia. While previous studies have shown that social information in the form of pain ratings influences pain perception^58^ and tends to dominate when presented alongside conditioned cues,^34,35^ our findings suggest this information can also alter pain perception when presented after the painful experience, which is in line with one previous study.^12^ Thus, verbal modeling could be effectively incorporated into therapeutic communication to mitigate nocebo effects and improve treatment outcomes. Informing patients about the source of pain judgments could engage social comparison and enhance learning through its effects on motivation, self-efficacy, or imitation of observed reactions.^57^

Consistent with previous findings,^41,55^ our results demonstrated the efficacy of counterconditioning in reducing nocebo hyperalgesia. In this procedure, the placebo—initially associated with high-intensity pain and responsible for triggering nocebo hyperalgesia—was later paired with a low-intensity pain. As a result, the original negative reaction was replaced by a more positive one, as participants learned that the same treatment now signaled a relatively more favorable experience—namely a sensation of pain relief—compared with when the treatment was not administered. These findings highlight the potential of counterconditioning to modify patients' maladaptive responses. However, as in previous studies, the procedure used here may not fully meet the criteria for counterconditioning, which involves pairing a stimulus previously associated with an aversive outcome with a new, positive one. Because the placebo was paired with the same outcome (pain) in both phases, the procedure could be considered reversal learning.

The attenuation effects remained stable across trials, demonstrating that the learning procedures can durably reduce nocebo hyperalgesia, extending prior findings on its stability^22,24^ and on the extinction of placebo hypoalgesia over time.^19,23^ Contrary to our hypotheses, no differences were observed in effectiveness across learning methods. Predicting such differences was challenging, as this study was novel and prior research primarily examined these methods during induction rather than attenuation.^34,35^ In addition, the relative effectiveness of these learning procedures in inducing placebo effects has not yet been tested within a single experiment. One possible explanation for the lack of differences is that each intervention introduced comparable prediction errors, which likely led to similar updating of participants' pain perceptions. Nevertheless, differences between procedures might emerge under untested conditions, such as delayed assessments or in chronic pain populations. This possibility is supported by expectation-related findings suggesting that the mechanisms underlying nocebo attenuation may vary across learning procedures.

In this study, expectancies fully mediated classically induced nocebo hyperalgesia, challenging research suggesting that awareness is not always necessary.^7–9^ While the cited studies used subtle, nonmedical placebos, such as visual cues, this study used a medical placebo (TENS), which may serve as a vehicle for preconditioned expectancies.^5,48^ These findings underscore the need to examine how contextual factors, including placebo type and administration method, affect expectancy formation.

Our first mediation analysis found no expectancy mediation during attenuation, but additional analysis conducted separately for each experimental group revealed indirect-only mediation for operant conditioning. Previous research shows that expectancies mediate placebo and nocebo effects induced by operant conditioning,^19^ verbal modeling,^47^ and classical conditioning.^23^ In our 2-stage procedure (nocebo induction followed by attenuation), cognitive demands related to updating prior associations may have limited participants' ability to accurately evaluate expectancies. Our finding that expectancies mediated attenuation only in the operant conditioning group—where explicit feedback was provided—is consistent with educational and cognitive psychology research showing that operant paradigms enhance attention and performance in discrimination tasks.^2,44^

In this study, longer response times were observed during placebo trials in both testing phases. Given that longer response times are associated with more difficult tasks,^59^ it seems that placebo trials may have increased cognitive load—participants may have engaged in greater cognitive monitoring or paid closer attention to their bodily sensations to verify whether the activated device influenced pain experience. This result aligns with evidence showing that reaction times in pain rating tasks can reflect metacognitive aspects of pain evaluation, with faster responses linked to greater confidence in pain judgments.^27^ Because the same response time pattern appeared in control groups, it can be assumed that the differences in response times were not due to nocebo hyperalgesia induction or attenuation. Nevertheless, these findings highlight the value of incorporating behavioral measures such as response times into placebo research, as they may reveal cognitive dynamics not fully captured by self-report and advance understanding of non-verbal pain assessment.

Although small effects on physiological measures (HR, SCL, SCR) were observed, no consistent pattern emerged. Similar studies show mixed findings, with some reporting no changes despite effects on pain^13^ or expectancy ratings,^40^ while others found effects on physiological responses.^23,39^ These inconsistencies, together with the complexity and sensitivity of physiological measures,^26^ highlight the need for further systematic research and make it difficult to determine whether observed changes result directly from experimental manipulation or from unrelated cues.

In this study, stress did not affect the induction or attenuation of nocebo hyperalgesia, despite previous links reported in the literature.^4,47,50^ This suggests that stress may not be essential for placebo effects, with cognitive factors such as expectancies playing a greater role. No significant correlations emerged between psychological traits and nocebo hyperalgesia, consistent with prior research,^32^ although the sample size may have limited detection of such associations.

This study is the first to compare verbal modeling, operant conditioning, and counterconditioning in reducing nocebo hyperalgesia within a single design. Verbal modeling and operant conditioning were tested against both a no-intervention control and a counterconditioning group, previously shown effective,^33,53^ mirroring randomized controlled trials.^51^ Booster trials initially maintained hyperalgesia, akin to partial reinforcement,^24^ yet all interventions reduced it. A medical placebo was used to enhance ecological validity, as these yield stronger effects than nonmedical placebos.^20^

Regarding limitations, this study was conducted on healthy volunteers, so further testing in clinical populations is needed. Pain was induced using electrocutaneous stimuli, limiting generalization to clinical pain. The sample size was calculated based on within-subject effects, whereas our primary hypothesis involved a between-within interaction, potentially resulting in insufficient power to detect the group × phase interaction.

Beyond the observational learning applied here, other modeling strategies (e.g., in-person or video observation) should be investigated, given the influence of social media on health.^29^ Latent learning, already shown effective,^22^ also remains underexplored in clinical contexts. To date, attenuation of nocebo effects has been tested only for those shaped by classical conditioning,^33,53^ so future research should examine whether effects induced by other methods are similarly attenuable.

From a procedural standpoint, the high number of pain stimuli used for nocebo induction and attenuation suggests the need to test for habituation or sensitization, followed by recalibration if necessary. Future studies could use extended testing to evaluate the durability of reversal effects and compare learning procedures with extinction. They could apply Bayesian hypothesis testing to formally integrate prior evidence and generate probabilistic statements regarding the relative efficacy of attenuation procedures.

In conclusion, this study provides new insights into eliminating nocebo hyperalgesia. As attenuation procedures showed comparable effectiveness, clinicians can select techniques based on convenience or individual patient response. Moreover, the last-used intervention may influence previous effects, which is clinically relevant for structuring the timing of pain management strategies—introducing an attenuating technique after a procedure that produces an undesirable effect may yield beneficial results.

## Conflict of interest statement

The authors have no conflicts of interest to declare.

## Supplementary Material

## Appendix A. Supplemental digital content

Supplemental digital content associated with this article can be found online at http://links.lww.com/PAIN/C459.
