# Supplementary material for: Verbal modeling, counterconditioning, and operant conditioning are effective in nocebo hyperalgesia attenuation
Source: Pain. 2026 Mar 4;167(6):1343–55. doi: 10.1097/j.pain.0000000000003934 (PMC13225248; doi:10.1097/j.pain.0000000000003934)
Supplement: Supplementary file 1 [file jop-167-1343-s001.pdf]

Supplementary Table A. Induction of nocebo hyperalgesia: Results of Welch's t-tests comparing merged experimental and merged control groups with and without outliers

t	df	$P_{one\text{-}sided}$	Cohen's d	SE Cohen's d
Total sample				
-1.522	99.113	0.066	-0.248	0.180
		Without outliers (N=3)		Note:
-1.818	102.604	0.036	-0.295	0.183
Without outliers and those who guessed the true aim of the study (N=18)				
-1.981	107.177	0.025	-0.332	0.189

Supplementary Table B1. Mean pain ratings across phases, trials and groups – total sample

Phase	Trial	Group	Mean	SD	N
Testing 1	Placebo trials	Operant	5,0000	1,39290	40
		Verbal modelling	4,8140	1,59300	43
		Counterconditioning	4,3504	1,48151	39
		Control (sham)	4,5159	0,94708	21
		Control (no-manipulation)	4,9792	1,42114	20
	Control trials	Operant	4,8083	1,27171	40
		Verbal modelling	4,5407	1,64704	43
		Counterconditioning	4,0769	1,46017	39
		Control (sham)	4,5476	1,10132	21
		Control (no-manipulation)	4,8125	1,29999	20
Testing 2 (first 4 trials)	Placebo trials	Operant	4,8250	1,18511	40
		Verbal modelling	4,9806	1,68127	43
		Counterconditioning	4,3077	1,75429	39
		Control (sham)	4,3968	1,31601	21

		Control (no-manipulation)	5,0208	1,59423	20
	Control trials	Operant	4,9167	1,04340	40
		Verbal modelling	4,8430	1,55363	43
		Counterconditioning	4,3568	1,74759	39
		Control (sham)	4,5437	1,39284	21
		Control (no-manipulation)	4,9375	1,39519	20
Testing 2 (last 4 trials)	Placebo trials	Operant	5,0708	1,11525	40
		Verbal modelling	4,8372	1,72199	43
		Counterconditioning	4,4359	1,81716	39
		Control (sham)	4,5119	1,58274	21
		Control (no-manipulation)	5,2750	1,41656	20
	Control trials	Operant	5,0688	1,26464	40
		Verbal modelling	4,8198	1,71661	43
		Counterconditioning	4,5769	1,72945	39
		Control (sham)	4,3333	1,41495	21
		Control (no-manipulation)	5,3042	1,47624	20

Supplementary Table B2. Mean pain ratings across phases, trials and groups – nocebo responders

**Phase**	**Trial**	**Group**	**Mean**	**SD**	**N**
Testing 1	Placebo trials	Operant	5,6435	1,49640	18
		Verbal modelling	4,9700	1,11073	25
		Counterconditioning	4,7875	1,41045	20
	Control trials	Operant	4,7917	1,44769	18
		Verbal modelling	4,2200	0,95981	25
		Counterconditioning	4,0167	1,35713	20
Testing 2 (first 4 trials)	Placebo trials	Operant	5,0556	1,14903	18

		Verbal modelling	5,0367	1,53284	25
		Counterconditioning	4,3208	1,50128	20
	Control trials	Operant	5,0972	1,17304	18
		Verbal modelling	4,6900	1,19957	25
		Counterconditioning	4,4167	1,59998	20
Testing 2 (last 4 trials)	Placebo trials	Operant	5,3056	1,16807	18
		Verbal modelling	4,8733	1,52755	25
		Counterconditioning	4,5167	1,59783	20
	Control trials	Operant	5,2083	1,41486	18
		Verbal modelling	4,8233	1,49562	25
		Counterconditioning	4,6667	1,33416	20

Supplementary Table C. Attenuation of nocebo hyperalgesia: Results of RM ANOVA with and without outliers

**Effect**	**Sum of Squares**	**df**	**Mean Square**	**F**	**p**
Phase	23,447	1	23,447	34,266	0,000
Phase * Group	3,530	2	1,765	2,580	0,084
Residuals	42,425	62	0,684		
Group	0,484	2	0,242	0,485	0,618
Residuals	30,911	62	0,499		
**Without outliers**					
Phase	16,079	1	16,079	35,396	0,000
Phase * Group	1,712	2	0,856	1,884	0,161
Residuals	27,256	60	0,454		
Group	1,051	2	0,525	1,282	0,285
Residuals	24,600	60	0,410		
**Without outliers and those who guessed the true aim of the study**					
Phase	15,407	1	15,407	31,555	0,000

Phase * Group	0,939	2	0,470	0,962	0,389
Residuals	26,366	54	0,488		
Group	1,184	2	0,592	1,415	0,252
Residuals	22,601	54	0,419		

Note: Analyses conducted on nocebo responders

Supplementary Figure A. Pain expectancies as a mediator of the nocebo attenuation effect (merged experimental groups).

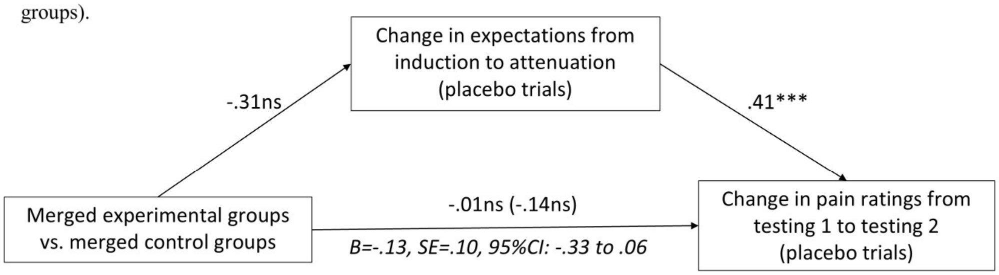

*Note: ***p<.001; ns – not significant; N=82; standardized regression coefficients (β) are presented; indirect effects are written in italics; total effects are presented in brackets; analysis was conducted on nocebo responders; R^2=.17, F (2, 79) =8.03; p<.001.*
